# Mechanisms for the α-Adrenoceptor-Mediated Positive Inotropy in Mouse Ventricular Myocardium: Enhancing Effect of Action Potential Prolongation

**DOI:** 10.3390/ijms24043926

**Published:** 2023-02-15

**Authors:** Shogo Hamaguchi, Ikue Morinou, Yuko Shiseki, Ayako Mikami, Maika Seki, Iyuki Namekata, Hikaru Tanaka

**Affiliations:** Department of Pharmacology, Faculty of Pharmacological Science, Toho University, Chiba 274-8510, Japan

**Keywords:** mouse ventricle, α-adrenergic receptor, L-type Ca^2+^ channel, action potential duration

## Abstract

Mechanisms for the α-adrenoceptor-mediated positive inotropy in neonatal mouse ventricular myocardium were studied with isolated myocardial preparations. The phenylephrine-induced positive inotropy was suppressed by prazosin, nifedipine, and chelerythrine, a protein kinase C inhibitor, but not by SEA0400, a selective Na^+^/Ca^2+^ exchanger inhibitor. Phenylephrine increased the L-type Ca^2+^ channel current and prolonged the action potential duration, while the voltage-dependent K^+^ channel current was not influenced. In the presence of cromakalim, an ATP-sensitive K^+^ channel opener, the phenylephrine-induced prolongation of action potential duration, as well as the positive inotropy, were smaller than in the absence of cromakalim. These results suggest that the α-adrenoceptor-mediated positive inotropy is mediated by an increase in Ca^2+^ influx through the L-type Ca^2+^ channel, and the concomitant increase in action potential duration acts as an enhancing factor.

## 1. Introduction

The sympathetic nervous system is the major regulator of cardiac function. It is involved in the acute and chronic control of myocardial growth and inotropy [1,2,3,4,5]. Noradrenaline released from sympathetic nerve endings regulates cardiac function not only via the β-adrenergic receptors, but also α-adrenergic receptors. While β-adrenergic receptor stimulation causes positive inotropy and chronotropy in most mammalian species, the response to α-adrenoceptor stimulation varies depending on the animal species [5]. α-adrenoceptor-mediated inotropy is positive in rats and rabbits, but not observed in dogs. In the adult mouse, α-adrenoceptor-mediated inotropy is negative in the right ventricle [6,7], but was reported to be positive in the left [8]. α-adrenoceptor-mediated positive inotropy was observed in the human right ventricle [9]. Various cellular mechanisms have been reported for α-adrenoceptor-mediated inotropy: the Ca^2+^ channel activation [10,11,12] and the K^+^ channel inhibition [13,14] for the positive inotropy and the Na^+^-Ca^2+^ exchanger (NCX) activation [15,16,17] and the decrease in Ca^2+^ sensitivity [18] for the negative inotropy. These results imply that the α-adrenoceptor-mediated inotropy consists of positive and negative components, and the balance between the positive and negative components determines the overall direction of inotropy.

α-adrenoceptor-mediated inotropy appears to be affected by action potential properties of the myocardium. In the diabetic rat ventricular myocardium, the β-adrenoceptor-mediated inotropy is impaired, and instead, an enhancement of α-adrenoceptor-mediated inotropy is observed [19,20]. In the diabetic mouse ventricular myocardium, the α-adrenoceptor-mediated negative inotropy was reduced [21]. In both cases, the action potential duration was prolonged, and in the case of the mouse, a causal relationship between action potential duration and inotropy was suggested. During the postnatal development of the mouse right ventricular myocardium, the α-adrenoceptor-mediated inotropy changes from positive to negative [6]. A similar developmental shift of inotropy from positive to negative was also observed with angiotensin II and endothelin I [22]. These changes occur concomitantly with a drastic developmental shortening of the action potential duration and changes in intracellular Ca^2+^ handling mechanisms [23]. We have reported that the negative inotropy in adult mice was caused by trans-sarcolemmal Ca^2+^ extrusion through the NCX, and was enhanced by the extremely short duration of the action potential [15,16]. On the other hand, the mechanisms for the positive inotropy in the neonatal mouse ventricle have not yet been clarified.

In the present study, we investigated the ionic mechanisms for the α-adrenoceptor-mediated positive inotropy in the neonatal mouse ventricle and further examined its relationship with the action potential duration.

## 2. Results

### 2.1. Measurement of Contractile Force

**Figure 1:**
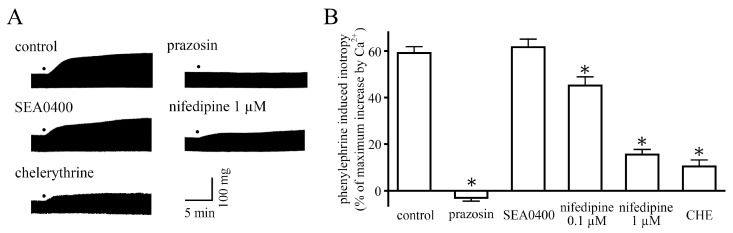
Effects of pharmacological interventions on the positive inotropic responses to phenylephrine. (**A**) Typical traces of the positive inotropy induced by phenylephrine in the absence and presence of prazosin, SEA0400, nifedipine, and chelerythrine (CHE). Dots indicate the application of 10 µM phenylephrine. (**B**) Summarized results for effects of the drugs on the positive inotropy of neonatal mice. Columns and vertical bars indicate the mean ± S.E.M. from 5–6 ventricular preparations. The asterisks indicated significant differences, *p* < 0.05, from corresponding control values, as evaluated by one-way ANOVA with Dunnett’s test.

In the neonatal mouse ventricular myocardium, phenylephrine (10 µM) induced a positive inotropy; this response is about 80% of the maximum response induced by phenylephrine [6]. The response was completely inhibited by prazosin (1 µM), an α_1_-adrenoceptor antagonist [24] (Figure 1). SEA0400 (10 µM), a selective NCX inhibitor [25], had no effect on the positive inotropy. Nifedipine (0.1 and 1 µM), an L-type Ca^2+^ channel inhibitor [26], induced a concentration-dependent inhibition of the positive inotropy. Chelerythrine (30 µM), a protein kinase C inhibitor [27], inhibited the positive inotropy.

### 2.2. Measurement of Membrane Currents

**Figure 2:**
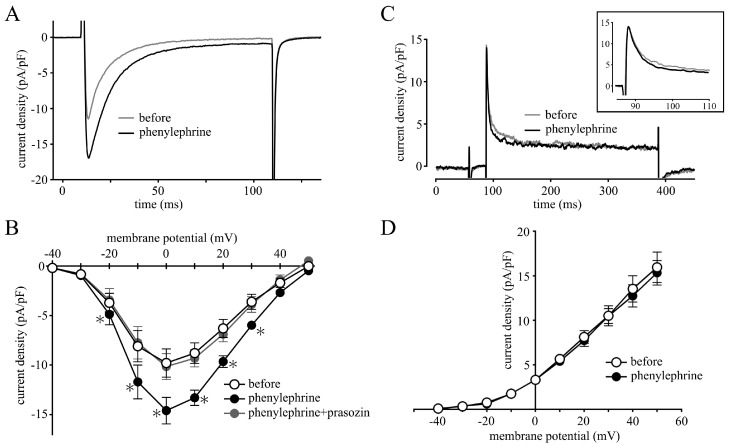
Effects of phenylephrine on the L-type Ca^2+^ current and voltage-dependent K^+^ current. Typical recording of the L-type Ca^2+^ current (**A**) and the K^+^ current (**C**) in the absence (gray) and presence (black) of 10 µM phenylephrine. The enlarged trace of the time axis was shown as an inset in (**C**). Current–voltage relationship of the L-type Ca^2+^ current (**B**) and the K^+^ current (**D**) obtained before (open circles) and after (closed circles) the application of 10 µM phenylephrine and after further application of 1 µM prazosin (gray circles). Symbols and vertical bars indicate the mean ± S.E.M. from 5 cardiomyocytes. The asterisks indicated significant differences, *p* < 0.05, from corresponding control values, as evaluated by one-way ANOVA with Dunnett’s test.

In the neonatal mouse ventricular cardiomyocytes, the amplitude of I_Ca-L_ was increased by 10 µM phenylephrine without changes in the shape of the current–voltage relationship (Figure 2A,B); the average peak inward current at 0 mV in the absence and presence of phenylephrine was −9.8 ± 1.4 and −14.6 ± 1.3 pA/pF, respectively (*n* = 5). Prazosin (1 µM) completely suppressed the increase in I_Ca-L_ induced by phenylephrine; the average peak inward current at 0 mV was −10.2 ± 1.3 pA/pF (*n* = 5). 

The amplitude of voltage-dependent K^+^ current was not influenced by 10 µM phenylephrine (Figure 2C,D); the average peak outward current at 50 mV in the absence and presence of phenylephrine was 16.0 ± 1.7 and 15.3 ± 1.4 pA/pF, respectively (*n* = 5). 

### 2.3. Measurement of Action Potentials

**Figure 3:**
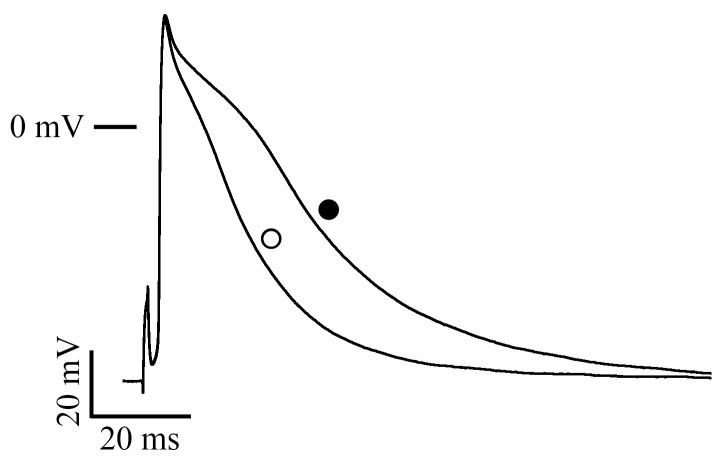
Effects of phenylephrine on the action potential configurations. Typical recording of action potential in the absence (open circle) and the presence (closed circle) of 10 µM phenylephrine.

In the neonatal mouse ventricular myocardium, phenylephrine (10 µM) prolonged the action potential duration at 20%, 50%, and 90% repolarization (APD_20_, APD_50_, and APD_90_, respectively) by 5.1 ± 0.5, 8.1 ± 0.8, and 27.4 ± 2.2 ms, respectively, with no change in other action potential parameters (Figure 3, Table 1). 

### 2.4. Effect of Changes in Action Potential Duration on the Positive Inotropy

**Figure 4:**
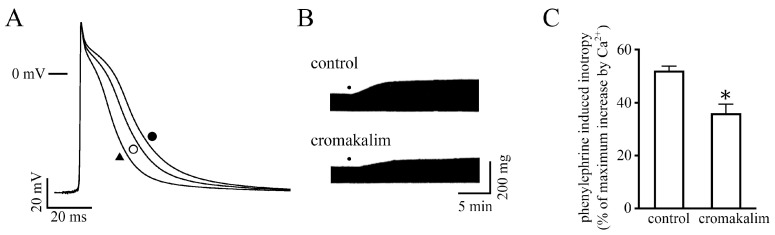
Effect of cromakalim on the phenylephrine-induced action potential duration prolongation and the positive inotropy. (**A**) Typical action potential recording in the absence (open circle) and presence (closed triangle) of 30 µM cromakalim and after further application of 10 µM phenylephrine (closed circle). (**B**) Typical traces of the positive inotropy in the absence and presence of 30 µM cromakalim. Dots indicate the application of 10 µM phenylephrine. (**C**) Summarized results for effects of the drugs on the positive inotropy. Columns and vertical bars indicate the mean ± S.E.M. from 8 ventricular preparations. The asterisk indicates a significant difference, *p* < 0.05, from corresponding values in control, as evaluated by Student’s *t*-test.

To examine whether the changes in action potential duration affect the positive inotropy, cromakalim, an ATP-sensitive K^+^ channel opener, was used. Cromakalim (30 µM) decreased the contractile force to 80.3 ± 2.7% of the value of its absence (*n* = 6) and shortened the APD_20_, APD_50_, and APD_90_ with no change in other action potential parameters (Figure 4A, Table 1). Thus, we considered that by combining phenylephrine and cromakalim, the effect of phenylephrine on the contractile force could be observed independently of its prolonging effect on the action potential duration. 

Both applications of cromakalim and phenylephrine prolonged the APD_20_, APD_50_, and APD_90_ by 2.3 ± 0.5, 1.8 ± 1.2, and 2.0 ± 3.8 ms from their absence, respectively (Figure 4A, Table 1). These prolongations were significantly smaller than those induced by phenylephrine alone (*p* < 0.05, evaluated by Student’s *t*-test). In the presence of cromakalim and phenylephrine, the positive inotropy was smaller than that induced by phenylephrine alone (Figure 4B,C).

## 3. Discussion

In the present study, we examined the mechanism for the α-adrenoceptor-mediated positive inotropy in the neonatal mouse ventricle. The mouse ventricular myocardium at this developmental stage has a poorly developed sarcoplasmic reticulum and lacks T-tubules [23]. Thus, the contraction of the neonatal ventricular myocardium is highly dependent on trans-sarcolemmal Ca^2+^ influx. In the present study, the inward current through the L-type Ca^2+^ channel, the major pathway for trans-sarcolemmal Ca^2+^ influx, was enhanced by α-adrenoceptor stimulation (Figure 2A,B). This was similar to the case in several other animal species [10,11,12,15]. Blockade of the L-type Ca^2+^ channel by nifedipine inhibited the α-adrenoceptor-mediated positive inotropy (Figure 1). These results indicate that α-adrenoceptor-mediated positive inotropy is mediated by an increase in the L-type Ca^2+^ channel current. 

Another possible pathway for trans-sarcolemmal Ca^2+^ influx is the NCX, which is known to be the major mechanism for trans-sarcolemmal Ca^2+^ efflux but may cause Ca^2+^ influx under certain conditions [28]. The NCX current was reported to be enhanced by α-adrenoceptor stimulation in several animal species including the adult mouse [15,16]. In the case of the neonatal mouse ventricle, the α-adrenoceptor-mediated positive inotropy was not affected by a selective NCX inhibitor, SEA0400, which indicated that the NCX was not involved (Figure 1). 

Concerning the signal transduction pathway, the α-adrenergic receptor is one of the Gq protein-coupled receptors, stimulating IP_3_ production and protein kinase C (PKC) activation. It was reported in rat ventricular cardiomyocytes that the phenylephrine-induced increase in L-type Ca^2+^ channel current was suppressed by chelerythrine, a PKC inhibitor [12]. This suggests that PKC is involved in the enhancement of the L-type Ca^2+^ channel current in the neonatal mouse ventricle. Inhibition of the inotropy by chelerythrine supports this view (Figure 1). However, the regulation of L-type Ca^2+^ channels by Gq-linked receptors and associated PKC activation is complex [29,30], and further research is needed for definitive conclusions.

Another possible mechanism for the α-adrenoceptor-mediated positive inotropy is inhibition of the K^+^ channel current. Decrease in the repolarizing K^+^ current results in prolongation of the action potential duration, which increases the time for Ca^2+^ influx through L-type Ca^2+^ channels and may cause positive inotropy. In rat and rabbit ventricles, α-adrenoceptor stimulation inhibits the transient outward K^+^ current, which is the major repolarization current in rat and mouse ventricles, resulting in the action potential prolongation and positive inotropy [13,14]. In the case of the rat, it was reported that α-adrenoceptor stimulation by methoxamine did not increase the L-type Ca^2+^ channel current, and the positive inotropy was attributed exclusively to the prolonged action potential duration [31], while other reports demonstrated increases in the L-type Ca^2+^ channel current [10,11,12]. In contrast, in the neonatal mouse ventricle, the K^+^ current was not influenced by phenylephrine (Figure 2B,D), excluding the contribution of K^+^-channel-mediated mechanisms in α-adrenoceptor-mediated prolongation of the action potential duration. The action potential prolongation observed after α-adrenoceptor stimulation is the result of increased inward current through L-type Ca^2+^ channels. 

The contraction of the neonatal mouse ventricle was sensitive to changes in action potential duration, similar to the case in the adult rat. As mentioned above, the α-adrenoceptor-mediated positive inotropy was accompanied by prolongation of action potential duration. Cromakalim, an ATP-sensitive K^+^ channel opener, shortened the action potential duration without affecting other action potential parameters and decreased the contractile force (Figure 4A, Table 1). In the presence of cromakalim, the phenylephrine-induced action potential prolongation and positive inotropy were significantly smaller than those in the absence of cromakalim (Figure 4B and C). Concerning the selectivity of cromakalim, it was reported that cromakalim decreased action potential duration and Ca^2+^ transient; these effects were fully canceled by glibenclamide, an ATP-sensitive potassium channel inhibitor [32]. The effect of cromakalim on the positive inotropy seemed unrelated to its off-target effects. These results suggest that the action potential duration serves as an enhancing factor for the α-adrenoceptor-mediated positive inotropy. 

The α-adrenoceptor-mediated inotropy in the mouse right ventricle changes from positive to negative during early postnatal development [6], which appears to be related to changes in action potential properties. In the adult mouse ventricle, although L-type Ca^2+^ channel current was increased by phenylephrine [15], as in the neonatal case (Figure 3), α-adrenoceptor-mediated positive inotropy was not observed [6,7]. The transient outward K^+^ current density in the adult mouse cardiomyocyte is higher than in the neonate, and the action potential is very short; α-adrenoceptor stimulation does not prolong the action potential duration [15]. These action potential properties are unfavorable for α-adrenoceptor-mediated positive inotropy, which involves Ca^2+^ influx through L-type Ca^2+^ channels. Lower repolarizing power in the neonatal mouse ventricle plays a permissive role in the action potential prolongation on α-adrenergic stimulation and favors positive inotropy. In addition, a negative inotropy is present in the adult mouse myocardium, which is mediated by the sarcolemmal NCX [15,16]. This electrogenic transporter pumps out one Ca^2+^ in exchange for three Na^+^, generating an inward current. α-adrenoceptor stimulation causes an enhancement of trans-sarcolemmal Ca^2+^ extrusion by the NCX leading to a decrease in intracellular Ca^2+^ and negative inotropy. A shorter action potential duration in the adult myocardium, which means a more negative membrane potential during elevated intracellular Ca^2+^ concentration, would accelerate Ca^2+^ extrusion by the NCX [28]. 

To what extent the present results obtained in the neonatal mouse apply in general should be carefully considered. Changes in the extent and direction of α-adrenoceptor-mediated inotropy have been reported in the myocardium under various pathological situations [1,2,3,4,5]. In the adult mouse right ventricle, α-adrenoceptor-mediated inotropy changes from negative in normal myocardium to positive in models of heart failure [33,34]. It has been reported that the action potential was prolonged during heart failure in mice [35]. Under diabetic conditions, an increase in α-adrenoceptor-mediated positive inotropy was observed in the rat [19,20], and a decrease in α-adrenoceptor-mediated negative inotropy was observed in the mouse [21]; both occurred concomitantly with the pathological action potential prolongation. In the human myocardium, the stimulation of the endothelin A (ET_A_) receptor, a Gq protein-coupled receptor similar to the α-adrenergic receptor, showed a positive inotropy in normal myocardium but negative in dilated cardiomyopathy [36]. There are reports about the prolonged action potential duration during heart failure in human and dog myocardium [37,38]. It is interesting if this is a mechanism to compensate for reduced contractility during heart failure. On the other hand, prolonged action potential duration may also cause arrhythmogenesis risks by producing a disturbance in intracellular calcium dynamics, which tends to occur in pathological conditions [39]. Therefore, whether the prolongation of action potential duration under pathological conditions serves as an effective compensatory mechanism is an interesting subject. Further studies in larger animals and human are awaited.

In conclusion, the α-adrenoceptor-mediated positive inotropy in neonatal mouse ventricles is mediated by an increase in Ca^2+^ influx through the L-type Ca^2+^ channel, and the concomitant increase in action potential duration acts as an enhancing factor.

## 4. Materials and Methods

### 4.1. General

All experiments were approved by the Ethics Committee of Toho University Faculty of Pharmaceutical Sciences (22-41-507) and performed in accordance with the Guiding Principles for the Care and Use of Laboratory Animals approved by The Japanese Pharmacological Society. The basic experimental setup and procedures were the same as those in our previous studies [6,7,15,16,21,22,23,40,41,42,43]. 

### 4.2. Measurement of Contractile Force

Whole ventricles were rapidly isolated from neonatal (0 to 2 days) ddY strain mice. Preparations were placed horizontally in a 20 mL organ bath containing the modified Ringer solution of the following composition (mM): NaCl 118.4, KCl 4.7, CaCl_2_ 2.5, MgSO_4_ 1.2, NaHCO_3_ 24.9, KH_2_PO_4_ 1.2, glucose 11.0 (pH 7.4 at 36 °C). The solution was gassed with 95% O_2_- 5% CO_2_ and maintained at 36 ± 0.5 °C. The preparations were driven by rectangular current pulses (1 Hz, 3 ms, 1.5 × threshold voltage) through a pair of platinum plate electrodes (field stimulation) generated from an electronic stimulator (SEN-3301; Nihon Kohden, Tokyo, Japan). The contractile force was recorded isometrically with a force-displacement transducer (TB-611T; Nihon Kohden) connected to a carrier amplifier (AP-621G; Nihon Kohden). The output was digitized by an A/D converting interface (Power Lab/4SP, AD Instruments, Chalgrove, UK) and analyzed by Chart 7 software (AD Instruments). The resting tension on each preparation was applied so that the muscle was stretched to the peak of its length/tension curve. The contractile force in the presence of phenylephrine was measured at 15 min after the addition of phenylephrine when it had reached a steady state. In all the experiments, experimental solutions contained propranolol (1 µM) to eliminate the β-adrenergic effects of phenylephrine.

### 4.3. Measurement of Action Potentials

Action potentials were recorded by standard microelectrode penetrations into an endocardial surface of the right ventricular free wall strips in neonatal mice. The glass microelectrodes filled with 3 M KCl had resistances of 20 to 30 MΩ. The preparations were driven by rectangular current pulses (1 Hz, 1 ms, 1.5 × threshold voltage) through a pair of platinum electrodes generated from the electronic stimulator (SEN-3301). The output of a microelectrode amplifier with high input impedance and capacity neutralization (MEZ8201, Nihon Kohden) was digitalized by the A/D converting interface (Power Lab/4SP) and analyzed by the Chart 7 software. The parameters measured were resting membrane potential (RP), overshoot (OS), amplitude (AMP), maximum rate of rise ($\overset{\cdot }{V}$_max_), and action potential duration at 20, 50, and 90% repolarization (APD_20_, APD_50_, and APD_90_, respectively).

### 4.4. Isolation of Mouse Ventricular Cardiomyocytes

Single ventricular myocytes were freshly prepared from the neonatal mouse by enzymatic dispersion using a “chunk” method, as described previously [23]. The hearts were rapidly excised in Tyrode’s solution of the following composition (mM): NaCl 143, KCl 5.4, CaCl_2_ 1.8, MgCl_2_ 1.0, NaH_2_PO_4_ 0.33, glucose 5.5, and HEPES 5.0 (pH adjusted to 7.4 with NaOH at 36 °C). The ventricles were placed in the Ca^2+^-free Tyrode’s solution for 10 min and then incubated in the Ca^2+^-free Tyrode’s solution containing a collagenase (10 mg/mL; Yakult, Tokyo, Japan) for 30 min at 37 °C. Thereafter, the collagenase was washed out with the Ca^2+^-free Tyrode’s solution and then stored in Kraftbrühe (KB) solution at 4 °C until use. Before voltage-clamp analysis, the ventricles were cut into small pieces in KB solution, and single cells were obtained using mechanical agitation.

### 4.5. Measurement of Membrane Currents

For the measurement of L-type Ca^2+^ current (I_Ca-L_) and voltage-dependent K^+^ current, a perforated patch clamp was carried out with amphotericin B to minimize the current rundown and maintain the adrenoceptor response. Amphotericin B was dissolved in dimethyl sulfoxide (100 μg/μL) and then was diluted with pipette solution using vortex to a final concentration of 200–400 μg/mL. 

The pipette solution for the K^+^ current contained (mM) KCl 100, KOH 40, MgCl_2_ 0.5, HEPES 10, EGTA 10, ATP-Mg 5, Na_2_ creatine phosphate 5, and GTP-Na 0.3 (pH 7.2 at 36 °C). The bathing solution was Tyrode’s solution as described above, containing 3 µM Nisordipine to eliminate I_Ca-L_. The K^+^ current was recorded by applying depolarizing pulses to -40 mV for 30 ms from a holding potential of −70 mV to eliminate Na^+^ current, followed by +50 mV for 300 ms at 35–36 °C. The current–voltage relationship of the K^+^ current was obtained using a series of test pulses between -40 mV and +50 mV in 10 mV increments.

The pipette solution for I_Ca-L_ contained (mM) CsCl 100, CsOH 40, MgCl_2_ 0.5, HEPES 10, EGTA 10, ATP-Mg 5, Na_2_ creatine phosphate 5, and GTP-Na 0.3 (pH 7.2 at 36 °C). The bathing solution contained (mM) NaCl 130, CsCl 20, CaCl_2_ 1.8, MgCl_2_ 1.0, NaH_2_PO_4_ 0.33, HEPES 5.0, glucose 5.5, and 4-aminopylidine 3.0 (pH 7.4 at 36 °C). I_Ca-L_ was recorded by applying depolarizing pulses to 0 mV for 100 ms, starting from a holding potential of −40 mV at 35–36 °C. The current–voltage relationship of I_Ca-L_ was obtained using a series of test pulses between −40 mV and +50 mV in 10 mV increments.

All membrane currents were recorded with the use of an Axopatch-200B amplifier (Molecular Device, San Jose, CA, USA). Data acquisition and analysis were performed with pCLAMP software 10 (Molecular Device). The resistance of filled electrodes ranged from 3 to 5 MΩ. 

### 4.6. Drugs and Chemicals

(R)-(-)-Phenylephrine hydrochloride, prazosin hydrochloride, nifedipine, chelerythrine, and cromakalim were purchased from Sigma-Aldrich (St. Louis, MO, USA), propranolol hydrochloride, and amphotericin B were purchased from FUJIFILM Wako Pure Chemical Corporation (Osaka, Japan). Nifedipine, prazosin, chelerythrine, and cromakalim were dissolved in dimethyl sulfoxide. Small aliquots were added to the extracellular solution to obtain the desired final concentration. The other chemicals were dissolved in distilled water. All other chemicals were commercial products of the highest available quality.

### 4.7. Statistics

All data are expressed as means ± standard error of the mean (S.E.M.). Statistical significance between means was evaluated by the paired *t*-test, Student’s *t*-test, or one-way analysis of variance (ANOVA) with Dunnett’s multiple comparisons. A *p*-value less than 0.05 was considered significant.

## Figures and Tables

**Table 1:** Changes in action potential parameters induced by pharmacological interventions.

	Control		Cromakalim		
	before	Phenylephrine	before	Cromakalim	+Phenylephrine
RP (mV)	−77.3 ± 0.8	−77.6 ± 1.0	−78.6 ± 0.8	−78.1 ± 0.3	−78.2 ± 0.4
OS (mV)	28.0 ± 2.2	28.3 ± 2.4	25.2 ± 3.4	25.9 ± 3.3	27.0 ± 2.5
AMP (mV)	105.3 ± 2.5	105.9 ± 2.6	103.8 ± 3.6	104.1 ± 3.5	105.2 ± 2.8
APD_20_ (ms)	7.0 ± 0.7	12.1 ± 1.0 *	6.6 ± 0.7	4.8 ± 0.7 *	8.9 ± 1.1 *
APD_50_ (ms)	14.9 ± 1.4	23.0 ± 2.0 *	18.2 ± 0.8	11.9 ± 0.7 *	20.0 ± 1.1
APD_90_ (ms)	40.5 ± 4.8	67.9 ± 5.0 *	47.7 ± 5.0	30.6 ± 1.6 *	49.7 ± 2.8
$\overset{\cdot }{V}$_max_ (V/s)	248.3 ± 21.5	244.9 ± 20.0	193.8 ± 13.0	202.5 ± 18.9	199.6 ± 19.5

Values are the mean ± S.E.M. from 4–5 ventricular preparations. Asterisks indicate significant differences from corresponding values in the absence of phenylephrine as evaluated by paired *t*-test or one-way ANOVA with Dunnett’s test.

## Data Availability

Not applicable.
